# Ultrafiltration combined with size exclusion chromatography efficiently isolates extracellular vesicles from cell culture media for compositional and functional studies

**DOI:** 10.1038/s41598-017-15717-7

**Published:** 2017-11-10

**Authors:** Birke J. Benedikter, Freek G. Bouwman, Tanja Vajen, Alexandra C. A. Heinzmann, Gert Grauls, Edwin C. Mariman, Emiel F. M. Wouters, Paul H. Savelkoul, Carmen Lopez-Iglesias, Rory R. Koenen, Gernot G. U. Rohde, Frank R. M. Stassen

**Affiliations:** 1 Department of Medical Microbiology, NUTRIM School of Nutrition and Translational Research in Metabolism, Maastricht University Medical Center, PO box 5800, 6202AZ Maastricht, The Netherlands; 2 Department of Respiratory Medicine, NUTRIM School of Nutrition and Translational Research in Metabolism, Maastricht University Medical Center, PO box 5800, 6202AZ Maastricht, The Netherlands; 3 Department of Human Biology, NUTRIM School of Nutrition and Translational Research in Metabolism, Maastricht University Medical Center, PO box 5800, 6202AZ Maastricht, The Netherlands; 4 Department of Biochemistry, Cardiovascular Research Institute Maastricht (CARIM), Maastricht University, Maastricht, The Netherlands, PO box 616, 6200 MD Maastricht, The Netherlands; 5 Department of Medical Microbiology & Infection Control, VU University Medical Center, Van Boechorststraat 7, 1081BT Amsterdam, The Netherlands; 6 Microscopy Core Lab, M4I Nanoscopy division, FHML, Maastricht University, Universiteitssingel 50, G0.201, 6229 ER Maastricht, The Netherlands; 7 Medical clinic I, Department of Respiratory Medicine, Goethe University Hospital, Frankfurt/Main, Germany

## Abstract

Appropriate isolation methods are essential for unravelling the relative contribution of extracellular vesicles (EVs) and the EV-free secretome to homeostasis and disease. We hypothesized that ultrafiltration followed by size exclusion chromatography (UF-SEC) provides well-matched concentrates of EVs and free secreted molecules for proteomic and functional studies. Conditioned media of BEAS-2B bronchial epithelial cells were concentrated on 10 kDa centrifuge filters, followed by separation of EVs and free protein using sepharose CL-4B SEC. Alternatively, EVs were isolated by ultracentrifugation. EV recovery was estimated by bead-coupled flow cytometry and tuneable resistive pulse sensing. The proteomic composition of EV isolates and SEC protein fractions was characterized by nano LC-MS/MS. UF-SEC EVs tended to have a higher yield and EV-to-protein rate of purity than ultracentrifugation EVs. UF-SEC EVs and ultracentrifugation EVs showed similar fold-enrichments for biological pathways that were distinct from those of UF-SEC protein. Treatment of BEAS-2B cells with UF-SEC protein, but not with either type of EV isolate increased the IL-8 concentration in the media whereas EVs, but not protein induced monocyte adhesion to endothelial cells. Thus, UF-SEC is a useful alternative for ultracentrifugation and allows comparing the proteomic composition and functional effects of EVs and free secreted molecules.

## Introduction

Extracellular vesicles (EVs) are secreted membrane vesicles that have emerged as important regulators of intercellular signalling. They are likely to contribute to homeostasis and disease via diverse functions, including the transfer of proteins and RNA between cells^1^. Adding to their importance, EVs are secreted by virtually all cells and are present in a variety of biological fluids, including blood^2^ and bronchoalveolar lavage fluid^3^. In a recent worldwide survey, ultracentrifugation was the most commonly applied EV isolation technique (81%) and washing of isolated EVs by ultracentrifugation the most common clean-up procedure (64%)^4^. However, ultracentrifuges are not available in all laboratories and ultracentrifugation is associated with several disadvantages. It has been found to co-isolate non-vesicle associated macromolecules^5^ and to cause EV aggregation^6^, which may bias the compositional and functional characterization of EVs. Recently, size exclusion chromatography (SEC) has been shown to provide high quality EV isolates from plasma^7^, which were sufficiently pure for Mass Spectrometric analysis^8^. Advantages of SEC include that no specialized equipment is required and that there is little risk for damage or aggregation of EVs as the technique relies on gravity flow. However, while SEC purifies EVs, it also dilutes them. This makes SEC unsuitable as a standalone technique for EV isolation from cell culture media, which are often less concentrated in EVs than plasma. Yet, cell culture media are the most commonly used starting material for EV isolation (83%)^4^, and robust and easy alternatives to isolation by ultracentrifugation are urgently needed. Two recent studies have provided evidence that concentrating conditioned media by ultrafiltration before SEC allows isolating EVs from dilute cell culture media^9,10^. These studies used a 100 kDa cut-off for ultrafiltration, which does not concentrate small secreted molecules. Yet, one of the major challenges in EV research is to establish the relative roles of EVs and the non EV-associated secretome in intercellular communication. A molecular weight cut-off of 10 kDa is expected to equally concentrate EVs and most free secreted signalling molecules. After separation using SEC, this should provide well-matched isolates of EVs and free protein from the same starting material for comparative studies. Here, we hypothesized that ultrafiltration followed by SEC (UF-SEC) allows isolating EVs from cell culture media with comparable or superior yield and purity as compared to ultracentrifugation. We also hypothesized that UF-SEC isolated EVs are suitable for proteomic analysis and that the EV-low protein-rich SEC fractions provide a well-matched control for studying differences in the proteomic composition and biological effects between EVs and the non-EV associated secretome. As a cell culture model, we used BEAS-2B bronchial epithelial cells that were either untreated or exposed to cigarette smoke extract (CSE) to induce pro-inflammatory activation^11^.

## Results

### Efficiency of EV isolation using ultrafiltration followed by SEC compared to ultracentrifugation

**Figure 1:**
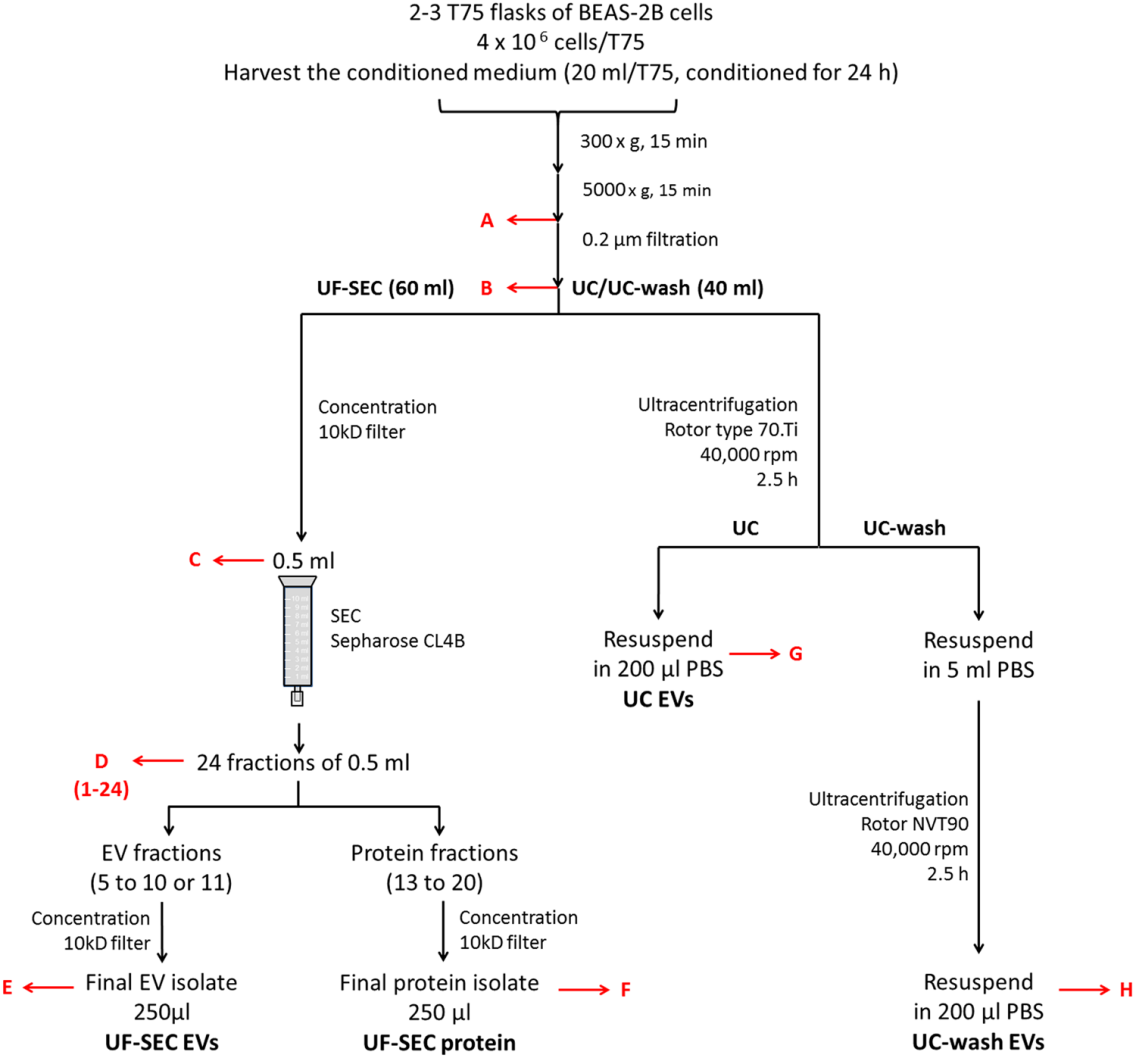
Flow chart of the EV isolation protocols. Red arrows indicate where samples were taken for determination of EV recovery based on CD63+ CD81+ bead-coupled flow cytometry. For nano LC/MS-MS, the isolation protocols were scaled up to 120 ml of cell culture medium as starting material. For UF-SEC, the 120 ml were concentrated by 10 kDa filtration and subsequently run over the SEC column in two aliquots of 0.5 ml. The EV containing fractions from both SEC runs (fractions 6 to 10 or 11) were then pooled and concentrated by 10 kDa filtration. For UC, the 120 ml were processed in 3 portions of 40 ml, corresponding to the maximal volume of the UC tubes.

The aim of this study was to evaluate whether ultrafiltration combined with size exclusion chromatography (UF-SEC) can serve as an easy and robust protocol to obtain high quality EV isolates for compositional analysis and functional studies. For this purpose, conditioned cell culture media were processed either by UF-SEC or, for comparison, by ultracentrifugation (UC) or ultracentrifugation including a wash step (UC-wash; illustrated in Fig. 1).

**Figure 2:**
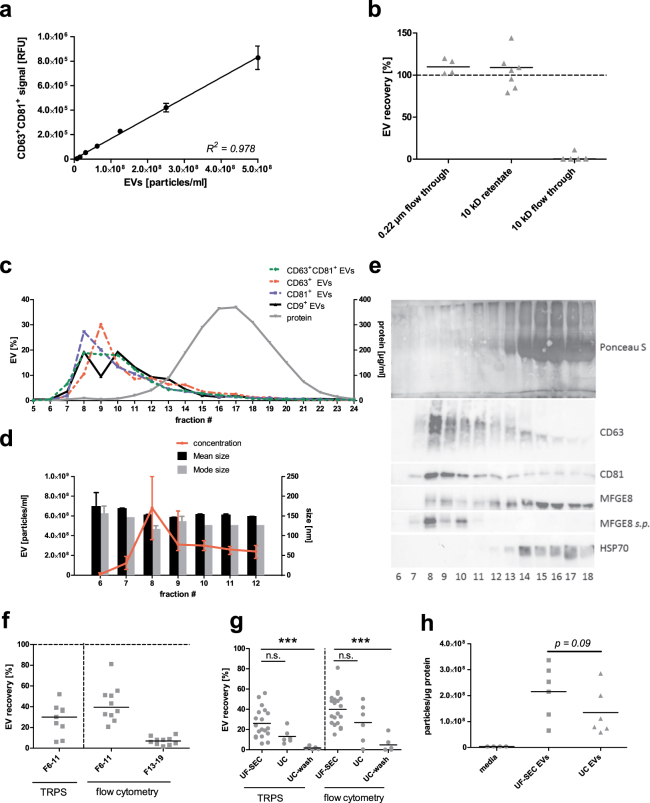
Evaluation of the isolation protocol. (**a**) A representative standard curve demonstrating that the CD63^+^CD81^+^ bead-coupled flow cytometry signal is proportional to the EV concentration determined by tuneable resistive pulse sensing (TRPS), allowing semi-quantitative EV measurements. (**b**) EV recovery after 0.22 µm filtration of cell-depleted media. The data represents flow cytometry samples A and B in Fig. 1. EV quantity in unfiltered cell-depleted media was set to 100%. (**c**) Protein concentration of the 24 SEC fractions determined by Bradford assay (µg/ml) and EV quantity determined by bead-coupled flow cytometry (relative fluorescent units; RFU) for the following combinations of antibodies (capture/detection): anti-CD63/anti-CD81, anti-CD63/anti-CD63, anti-CD81/anti-CD81, anti-CD9/anti-CD9 (sampling step D indicated in Fig. 1). Graph shows medians without error bars, n = 3. (**d**) EV mode size, mean size and concentration for SEC fractions 6–12 as determined by TRPS. The graph shows the median and range, n = 3. (**e**) Top: Ponceau S total protein staining of SEC fractions 5 to 18; bottom: Western blot of SEC fractions 5 to 18 for EV marker proteins CD63, CD81, MFGE8 and HSP70. For the lane MFGE8 *s.p*., a standardized protein concentration of 5 µg was loaded for each fraction. For all other stainings, whole fractions were precipitated and loaded on the gel without standardization of the protein content. (**f**) EV recovery in the final UF-SEC EV isolate (Fractions 6–11) and the final UF-SEC protein isolate (Fractions 13–19) as determined by TRPS or CD63^+^CD81^+^ flow cytometry. The EV quantity in conditioned culture medium has been set as 100%. (**g**) Comparison of the EV recovery for UF-SEC, UC or UC-wash, determined by TRPS (overall p-value = 0.0005) and CD63^+^CD81^+^ bead-couple flow cytometry (overall p-value = 0.0009; sampling steps E, G and H, respectively). Data was analysed using the Kruskall Wallis test followed by Dunn’s post-test. ***p < 0.001. (**h**) The EV-to-protein rate of purity base on TRPS and Bradford measurements of conditioned cell culture media, UF-SEC EVs and UC EVs. The statistical difference between UF-SEC EVs and UC EVs was assessed using the Mann-Whitney U test.

First, we established that CD63^+^CD81^+^-bead coupled flow cytometry can be applied for semi-quantitative assessment of EV recovery, as the flow cytometry signal is linear over a broad range of EV concentrations for a serial dilution of isolated EVs (Fig. 2a). There was no loss of CD63^+^CD81^+^ EVs after 0.22 µm filtration of the conditioned media (Fig. 2b). Moreover, the recovery of CD63^+^CD81^+^ EVs in the 10 kDa filter retentates was complete, while virtually no EVs were detectable in the flow through (Fig. 2b). The 10 kDa filter retentates (0.5 ml) were run over a sepharose CL-4B SEC column and, per run, 24 fractions of 0.5 ml were collected. Protein elution was assessed by Bradford assay or Ponceau S staining and EV elution by tuneable resistive pulse sensing (TRPS). Moreover, elution of several EV marker proteins was assessed by bead-coupled flow cytometry and immunoblotting. By Bradford assay, protein showed a bimodal elution pattern with a minor peak at fraction 8 (10 µg/ml) and a major peak at fraction 15 (389 µg/ml; Fig. 2c, detail in Supplementary Figure S1). Bead-coupled flow cytometry revealed slightly different elution patterns for CD63^+^CD81^+^, CD63^+^, CD81^+^ and CD9^+^ EVs (Fig. 2c, detail in Supplementary Figure S1). Yet, the peak elution of EVs coincided with the minor protein elution peak from fractions 7 to 10 irrespective of the detected EV marker protein (Fig. 2c, Supplementary Figure S1). TRPS further confirmed that the EV peak elution was in fraction 8 and showed that EV size was identical throughout fractions 6 to 12 (Fig. 2d). Using Ponceau S staining, total protein remained virtually undetectable until fraction 12, whereas immunoblots revealed the presence of the EV marker proteins CD63, CD81 and MFGE8 starting at fraction 7 and with a peak in fraction 8 (Fig. 2e). Of note, CD63 and CD81 were specifically enriched in the early fractions, whereas MFGE8 was detected at a similar intensity in all fractions (Fig. 2e). However, when the amount of protein loaded per fraction was standardized, MFGE8 was only detectable in fractions 7 to 10, suggesting a relative enrichment in the EV fractions (Fig. 2c, lane MFGE8 *s.p*.). Unexpectedly, HSP70, which is also considered an EV marker protein, was detected in the protein-rich fractions 13 to 18, rather than co-eluting with the other EV marker proteins (Fig. 2e). This questions the suitability of HSP70 as a universal and specific EV marker protein. Uncropped images of all immunoblots are available in Supplementary Figure S2.

The EV-enriched, protein-low (6 to 10 or 11) and the protein-enriched, EV-low (14–19) SEC fractions were concentrated on 10 kDa centrifuge filters to obtain EV and protein concentrates, respectively. The median EV recovery in the final UF-SEC EV isolate was 40% according to CD63^+^CD81^+^ bead-coupled flow cytometry, while 7% of EVs was lost in the final protein concentrate (Fig. 2f). By TRPS, the median recovery of EVs sized 80–250 nm was 30% (Fig. 2f). The EV recovery of the UF-SEC protocol was compared to that of UC or UC-wash. TRPS and CD63^+^CD81^+^ bead-coupled flow cytometry both found that EV recovery was higher for UF-SEC than for UC and UC-wash. For UC-wash, this was statistically significant (Fig. 2g). The purity of an EV isolate can be estimated by dividing the EV concentration of the sample by its protein concentration^7^. UF-SEC EVs had a slightly higher EV-to-protein rate of purity compared to UC EVs, although this was not statistically significant (Fig. 2h).

Taken together, UF-SEC efficiently concentrates EVs from cell culture media and separates them from the bulk of free protein. The yield and purity of these UF-SEC EVs tend to be superior to those of UC-EVs isolated using ultracentrifugation settings as optimized by Cvjetkovic *et al*.^12^.

### Characterization of the isolated EVs

**Figure 3:**
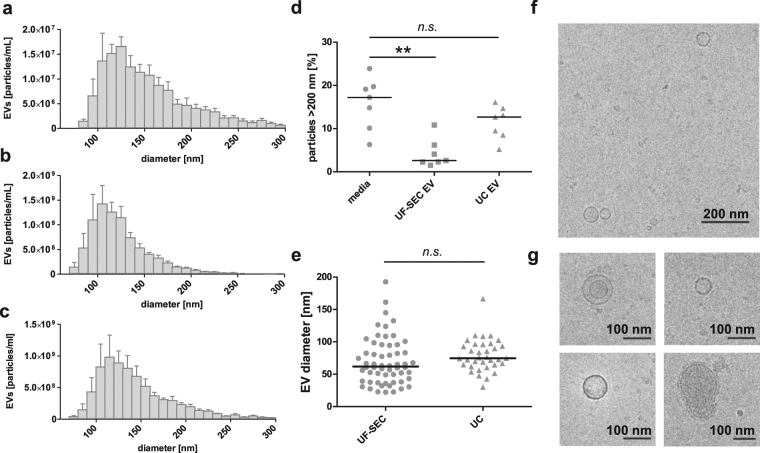
Characterization of EVs in unfiltered conditioned media, UF-SEC EVs and UC EVs. (**a**) Size distribution histogram of the EVs in (**a**) unfiltered conditioned media (**b**) UF-SEC EV isolates and (**c**) UC EV isolates as determined by TRPS. Graph shows the mean ± SEM, n = 5. (**d**) The percentage of particles >200 nm in conditioned media, UF-SEC EVs and UC EVs according to TRPS. The bar indicates the median diameter. **p 0.001–0.01 (**e**) Size distribution of EVs in UF-SEC and UC isolates based on cryo-TEM. The bar indicates the median diameter. (**f**) Low magnification and (**g**) detail cryo-TEM recording of UF-SEC isolated EVs from BEAS-2B.

EV size and morphology were assessed by TRPS and cryo-TEM. TRPS revealed that the EV size distribution was similar for EVs in unfiltered conditioned media (Fig. 3a), UF-SEC EVs (Fig. 3b) and UC EVs (Fig. 3c). For all three sample types, the vast majority of EVs ranged between 80 and 250 nm in diameter, with mode sizes between 105 and 125 nm. Although the UF-SEC and UC protocols both include a 0.22 µm filtration step, only UF-SEC EVs, but not UC-EVs contained significantly less particles larger than 200 nm compared to the unfiltered media (Fig. 3d), suggesting that aggregation may occur for the ultracentrifugation protocol. According to cryo-TEM, both UF-SEC EVs and UC EVs contained very small EVs below the detection limit of the qNano (<80 nm). The EV diameters were similar for UF-SEC EVs and UC EVs with medians of 61.9 and 74.6 nm, respectively (Fig. 3d). Representative low and high magnification cryo-TEM images of UF-SEC EVs are shown in Fig. 3e and f. We have previously published comparable cryo-TEM images of UC EVs^13^.

**Figure 4:**
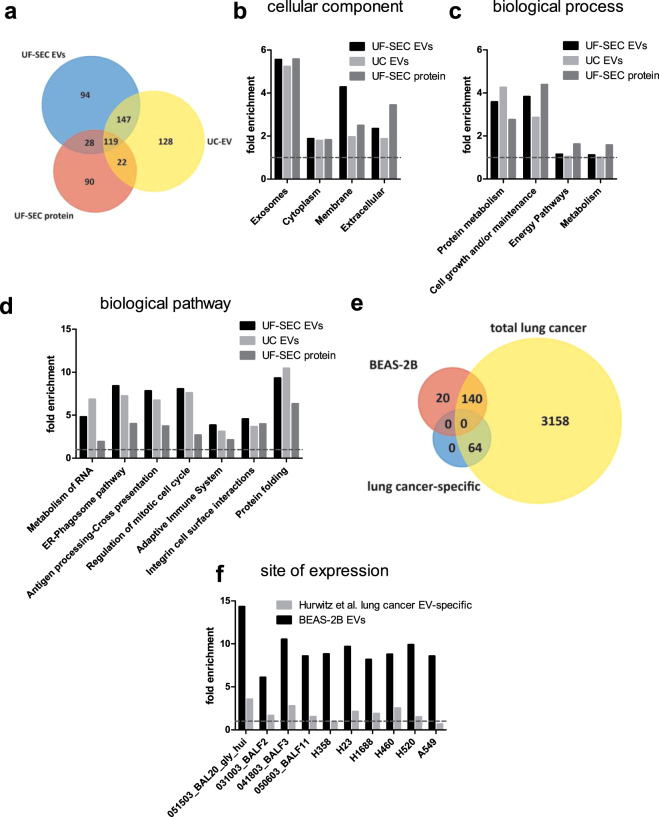
Proteomic characterization of UF-SEC EVs, UC EVs and UF-SEC protein from BEAS-2B cells. (**a**) Venn diagram of the proteins identified for UF-SEC EVs, UC EVs and UF-SEC protein. (**b**) Functional enrichment analysis for cellular component GO terms. (**c**) Functional enrichment analysis for biological process GO terms. (**d**) Functional enrichment analysis for manually curated biological pathways. (**e**) Venn diagram of proteins identified in UF-SEC EVs and UC EVs but not UF-SEC protein from BEAS-2B cells (BEAS-2B EVS) compared to the total lung cancer EV associated proteome and the lung cancer specific proteome according to Hurwitz *et al*.^16^. (**f**) Functional enrichment analysis for the site of expression for the BEAS-2B EV proteome and the lung cancer-specific proteome proposed by Hurwitz *et al*.^16^.

Proteomic analysis was performed by nano LC-MS/MS for UF-SEC EVs, UC EVs or UF-SEC protein fractions. Proteins with a Score Sequest HT >10 were considered as identified with high confidence. For the UF-SEC EVs these were 388 proteins, for UC EVs 421 and for UF-SEC protein 264 (Supplementary Table S1). Figure 4a shows a Venn diagram of the identified proteins. One hundred and nineteen proteins overlapped between all three datasets, and 147 between UF-SEC EVs and UC EVs, whereas the overlap between each individual type of EV isolate and the free secreted protein was relatively small (28 and 22 proteins for UF-SEC EVs and UC EVs, respectively). Table 1 shows 34 exosomal marker proteins compiled by de Menezes-Neto *et al*.^8^ and their respective expression in the UF-SEC EV, UC EV and UF-SEC protein isolates. Strikingly, most of these proposed exosomal markers were detected in all three datasets. Only Annexin A5, Annexin A6, CD63, CD81, CD9, MFGE8, PDCD6IP/Alix and syntenin-1 were identified in both UF-SEC EVs and UC EVs, but not in UF-SEC protein, suggesting that these may be the most specific among the 34 proposed exosome markers. Of note, HSP70 (*HSPA8*) was identified with a higher number of peptide spectral matches (#PSM) in the UF-SEC protein than in either UF-SEC EVs or UC EVs, in line with our finding that immunoblots stained positive for HSP70 in fractions 13 to 18 rather than in the early EV-fractions (Fig. 2e). Rab GTPases are enzymes that regulate membrane transport and fusion processes in the endosomal compartment that exosomes are derived from^14^. For UF-SEC EVs and UC EVs, but not UF-SEC protein, several Rab GTPases were identified (Supplementary Table S2). In all three sample types, most proteins considered as markers for contamination from intracellular organelles^15^ were absent, except histones and the endoplasmic reticulum associated protein grp94 (Table 2).

**Table 1:** List of EV marker proteins compiled by de Menezes-Neto *et al*.^9^ and their presence or absence in EVs isolated from conditioned media of BEAS-2B cells using UF-SEC. The number of peptide spectral matches (#PSM) gives an estimation of the relative protein abundance.

EV marker proteins	Gene symbol	UF-SEC EVs		UC-EVs		UF-SEC protein	
		identified	#PSM	identified	#PSM	identified	#PSM
Actin, cytoplasmic 1	ACTB	yes	79	yes	291	yes	297
Fructose-bisphosphate aldolase A	ALDOA	yes	13	yes	65	yes	108
Annexin A2	ANXA2	yes	42	yes	76	yes	6
Annexin A5	ANXA5	yes	12	yes	10	no	0
Annexin A6	ANXA6	yes	32	yes	33	no	0
CD63 antigen	CD63	yes	4	yes	5	no	0
CD81 antigen	CD81	yes	2	yes	9	no	0
CD82 antigen	CD82	yes	3	no	0	no	0
CD9 antigen	CD9	yes	8	yes	13	no	0
Cofilin-1	CFL1	yes	12	yes	18	yes	38
Clathrin heavy chain 1	CLTC	yes	142	yes	324	yes	4
Elongation factor 1-alpha 1	EEF1A1	yes	20	yes	56	yes	25
Ezrin	EZR	yes	22	yes	10	yes	20
Fatty acid synthase	FASN	yes	76	yes	274	yes	3
Glyceraldehyde-3-phosphate dehydrogenase	GAPDH	yes	26	yes	127	yes	43
Rab GDP dissociation inhibitor beta	GDI2	yes	3	yes	2	yes	19
Heat shock cognate 71 kDa protein	HSPA8	yes	49	yes	30	yes	126
Lactadherin	MFGE8	yes	22	yes	40	no	0
Moesin	MSN	yes	24	yes	9	yes	19
Programmed cell death 6-interacting protein	PDCD6IP	yes	21	yes	12	no	0
Phosphoglycerate kinase 1	PGK1	yes	15	yes	31	yes	113
Pyruvate kinase	PKM2	yes	51	yes	180	yes	86
Peroxiredoxin-1	PRDX1	yes	4	yes	14	yes	28
Ras-related protein Rap-1b	RAP1B	yes	8	yes	3	no	0
Radixin	RDX	yes	12	no	0	no	0
Transforming protein RhoA	RHOA	yes	9	yes	5	no	0
Rho-related GTP-binding protein RhoC	RHOC	no	0	yes	2	yes	1
Syntenin-1	SDCBP	yes	8	yes	15	no	0
Tumour susceptibility gene 101 protein	TSG101	no	0	no	0	no	0
14-3-3 protein beta/alpha	YWHAB	yes	15	yes	11	yes	26
14-3-3 protein epsilon	YWHAE	yes	11	yes	11	yes	16
14-3-3 protein gamma	YWHAG	yes	8	yes	17	yes	18
14-3-3 protein theta	YWHAQ	yes	14	yes	8	yes	19
14-3-3 protein zeta/delta	YWHAZ	yes	11	yes	9	yes	40

**Table 2:** List of contamination marker proteins (adapted from^13^) and their presence or absence in UF-SEC EVs, UC EVs or UF-SEC protein.

Contamination marker proteins	Gene symbol	Origin of contamination	UF-SEC EVs	UC-EVs	UF-SEC protein
endoplasmin/grp94	HSP90B1	ER	yes	yes	yes
calnexin	CANX	ER	no	no	no
130 kDa cis-Golgi matrix protein	GM130	Golgi	no	no	no
cytochrome c1	CYC1	mitochondria	no	no	no
histones	HIST*H*	Nucleus	yes	yes	yes
protein argonaut	AGO-*	argonaut/RISC complex	no	no	no

Next, functional enrichment analysis of the proteomics data was performed for cellular component gene ontology (GO) terms. Both UF-SEC EVs and UC-EVs showed a more than 5-fold enrichment for the GO term *exosomes* (Fig. 4b). However, the UF-SEC protein dataset was similarly enriched for *exosomes*, thus questioning the specificity of the finding (Fig. 4b). All three datasets were similarly enriched for *cytoplasm*. Yet, UF-SEC EVs were most prominently enriched for *membrane*, whereas UF-SEC protein was most strongly enriched for *extracellular*, suggesting that there was an efficient separation of membrane-associated and free secreted proteins by UF-SEC (Fig. 4b). Functional enrichment analysis was also performed for biological process GO terms (Fig. 4c) and for manually curated biological pathways (Fig. 4d). For most biological processes and pathways, the fold enrichment was similar for UF-SEC EVs and UC EVs, but distinct from UF-SEC protein. This implies that EVs may exert distinct functions compared to free secreted proteins. EV-enriched biological pathways included *metabolism of RNA*, *protein folding*, *regulation of mitotic cell cycle* and several immunity related pathways, such as *ER-phagosome pathway, antigen processing – cross presentation* and *adaptive immune system*.

We then aimed to determine whether BEAS-2B derived EVs contain proteins that are specific for lung epithelium-derived EVs. Hurwitz *et al*. have recently analysed the proteomic composition of EVs derived from 60 cancer cell lines, including 9 of pulmonary epithelial origin^16^. They identified 3362 unique proteins that were expressed in at least one of the 9 lung cancer cell lines. These comprised 64 proteins that were absent in EVs from the 51 non-lung cancer cell lines. Here, we included 160 proteins that were detected in both UF-SEC EVs and UC EVs, but not in UF-SEC protein. We tested the overlap of these BEAS-2B EV-associated proteins with either the total lung cancer EV proteome or the lung-cancer specific proteins according to Hurwitz *et al*. While 140 of the 160 BEAS-2B EV proteins overlapped with the lung cancer EV proteome identified by Hurwitz *et al*., none of them overlapped with the 64 lung cancer EV-specific proteins (Fig. 4e). However, in a functional enrichment analysis for the site of expression, the BEAS-2B EVs showed a much stronger fold-enrichment for lung epithelial-related sites of expression than the 64 proteins that were proposed as lung cancer EV-specific by Hurwitz *et al*. (Fig. 4f). Thus, the BEAS-2B EV dataset contained no individual lung epithelial-specific proteins, but a complex protein signature that indicates their origin.

Taken together, our UF-SEC protocol yields small to very small EVs expressing exosomal marker proteins. Moreover, our data suggests that besides universal EV-associated proteins, EVs also carry a protein signature that may be specific for their cell of origin.

### Functional analysis

**Figure 5:**
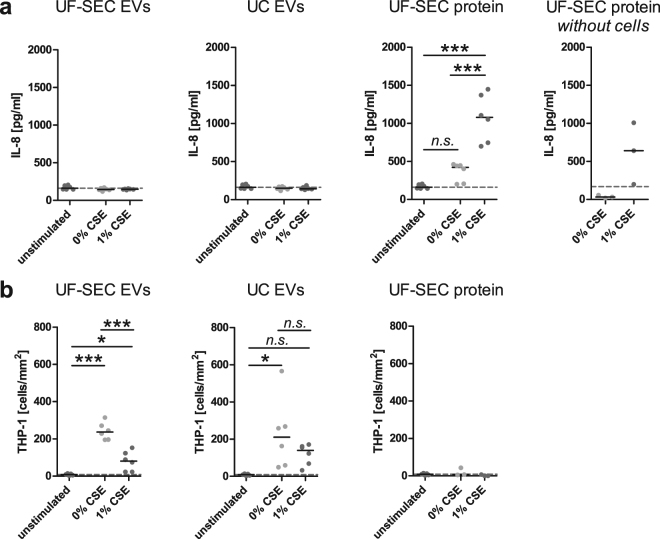
Differential functional effects of EVs and free secreted protein. Effect of the EVs obtained by UF-SEC or the protein fractions from unexposed (0% CSE) or CSE-stimulated BEAS-2B cells on (**a**) the IL-8 secretion of naïve BEAS-2B cells and (**b**) adhesion of THP-1 monocytes to HUVECs (overall p-value < 0.0001). Data was analysed by the Kruskall Wallis test followed by Dunn’s posthoc test. *p = 0.01-0.05, and ***p < 0.001 compared to the unstimulated control.

Finally, we aimed to assess whether UF-SEC is a suitable isolation method for comparing biological effects of EVs and the non-EV associated secretome. As Moon *et al*. have previously found that EVs from BEAS-2B bronchial epithelial cells stimulated with cigarette smoke extract (CSE) enhance IL-8 release from naïve BEAS-2B cells^11^, we first determined the effect of UF-SEC EVs, UC EVs and UF-SEC protein from unexposed or CSE-exposed BEAS-2B cells on naïve BEAS-2B cells. Cells were treated with 10^8^ EVs/ml or with volume-matched protein isolates from the UF-SEC protocol. After 48 h, supernatants were harvested and IL-8 concentrations determined using ELISA. In contrast to the observations by Moon *et al*., only the UF-SEC protein fractions, but neither UF-SEC EVs nor UC EVs resulted in increased IL-8 release (Fig. 5a). To test whether UF-SEC protein truly induced IL-8 secretion in naïve BEAS-2B cells, we assessed the IL-8 content of UF-SEC protein samples that were incubated for 48 h without cells. Figure 5a (UF-SEC protein without cells) shows that a substantial amount of IL-8 was detected in these cell-free samples, suggesting that the elevated IL-8 concentrations after stimulation of BEAS-2B cells with UF-SEC protein largely result from a carryover of IL-8 protein. We also assessed the effect of UF-SEC EVs, UC EVs and UF-SEC protein on adhesion of THP-1 monocytes to human umbilical cord endothelial cells (HUVECs). For this purpose, HUVECs were treated with 7.5 × 10^7^ EVs/ml or volume-matched protein isolates for 72 h. After removal of the EV containing medium, adhesion of THP-1 monocytes to the HUVECs was assessed in a flow chamber. Only UF-SEC EVs and UC EVs from control and CSE-stimulated BEAS-2B cells, but not the protein isolates resulted in adhesion of the monocytes (Fig. 5b). While results were comparable for UF-SEC EVs and UC EVs, the variance was greater for results obtained with the UC EVs (coefficient of variation 83.8% for 0% CSE UC EVs and 19.0% for 0% CSE UF-SEC EVs).

Thus, UF-SEC EVs yielded similar, but more reproducible results in our functional assays than UC EVs. Moreover, UF-SEC isolation provides well-matched EVs and non-EV-associated secreted protein samples, allowing comparative functional studies.

## Discussion

In this study, we established UF-SEC as an easy and robust method for obtaining EV isolates from cell culture media with sufficient yield and purity for compositional and functional characterization. In 2014, Böing *et al*. have demonstrated that SEC using 10 ml sepharose CL-2B columns efficiently isolates EVs from plasma^7^. However, the 10 ml sepharose CL-2B columns are only suitable for small sample volumes of about 0.5 ml. As cell culture media are relatively dilute in EVs, large volumes are required for EV isolation (often >100 ml^4^). Therefore, protocols have been developed where media are concentrated by ultrafiltration prior to SEC.^9,10^. Unfortunately, neither the media volumes before and after ultrafiltration, nor the size of the SEC column were reported in these studies. Here, we aimed to report our protocols in a detailed and transparent way to allow easy reproduction of our methodology by other research groups.

The majority of EVs in unfiltered media of the bronchial epithelial cells used in this study were between 80 and 250 nm in diameter as estimated by TRPS. To specifically concentrate and purify these relatively small EVs, we introduced a 0.22 µm filtration step, chose a small molecular weight cut-off for ultrafiltration and used a SEC matrix expected to efficiently separate small EVs from free protein. Instead of the 100 kDa filters used for media concentration in previous studies^9,10^, we used 10 kDa filters, which resulted in complete EV recovery in the concentrate. Similarly, Vergauwen *et al*. have recently demonstrated that EVs can be concentrated with complete recovery on 10 kDa filters, whereas recovery was only 40% for 100 kDa filters^17^. Importantly, they have also shown that recovery was best with the regenerated cellulose Amicon Ultra Centrifugal filters from Millipore that were also used in this study, while EV yield was highly inferior when using ultrafiltration systems with different filtration membrane materials^17^. The matrix of our SEC columns was sepharose CL-4B, which has a smaller size exclusion limit than the classically used sepharose CL-2B (42 vs. 75 nm)^18^. Using this matrix, all particles larger than 42 nm are expected to elute in early fractions, while smaller particles and molecules are delayed. This putatively provides a better separation of small EVs from free protein than matrices with a larger size exclusion limit. This is supported by the finding of Baranyai *et al*. that sepharose CL-4B provides a better separation of EVs from bovine serum albumin than sepharose CL-2B^19^. The improved separation does not appear to result in decreased yield as EV recovery in the early protein-low fractions is similar for sepharose CL-4B (40%, this study) and CL-2B (43%,^7^).

All in all, the combination of 0.22 µm filtration, concentration on a 10 kDa filter and CL-4B SEC in our isolation protocol yielded very small EVs (median size by cryo-TEM 61 nm, mode size by TRPS 105 nm). According to proteomics analysis, these EVs expressed several exosome marker proteins, including the tetraspanins CD63, CD81 and CD9, MFGE8 and several Rab GTPases that are suggestive of endosomal origin^14^. Moreover, most proteins considered to be contamination markers were absent, except for grp94 and several histones. However, grp94 currently has 109 entries in the EVpedia database^20,21^, as compared to 103 entries for the most commonly used exosome marker protein CD63. This suggests that, in contrast to the current consensus, grp94 may truly occur associated with EVs. Moreover, histones have been proposed to be sorted selectively into EVs by two independent studies^22,23^, questioning their categorization as contamination markers. Thus, our UF-SEC protocol yields small EVs with exosome properties with sufficient purity for proteomic studies.

As UC is currently the most widely applied and accepted technique for EV isolation^4^, we also compared the efficiency of our UF-SEC protocol to UC. Both, the EV-yield and the EV-to-protein rate of purity were similar for UF-SEC and UC, but tended to be superior for UF-SEC. Similarly, Mol *et al*. did not find any difference between SEC-isolated and UC-isolated EVs in terms of protein content and EV yield^10^. In contrast, Nordin *et al*. have reported a 5-fold higher EV-yield for UF-SEC than for UC^9^. This discrepancy may arise from divergences in the details of either the UF-SEC or the UC protocols that were applied in the different studies. Proteomics characterization revealed that the proteomes of UF-SEC EVs and UC EVs overlapped to a similar extent with non-EV associated secreted proteins (UF-SEC protein). This further supports that there is no clear difference in EV-to-protein purity between the two techniques. Additionally, bovine serum albumin (BSA) was the most abundantly identified protein in both UF-SEC EVs and UC EVs (Supplementary Table S1), highlighting that contamination with soluble proteins still occurs for both isolation techniques. However, UF-SEC EVs were more strongly enriched for the cellular component *membrane* than UC EVs (4.3-fold vs. 2.0-fold), suggesting a higher enrichment in membrane vesicles. In biological pathway analysis, UF-SEC EVs and UC EVs showed a similar pattern of enrichment that was distinct from the UF-SEC protein fractions. Pathways that were enriched in both types of EV isolates included maintenance functions such as *metabolism of RNA*, *regulation of mitotic cell cycle* and *protein folding*, as well as several pathways related to immunity, including *ER-phagosome pathway*, *antigen processing – cross presentation* and *adaptive immune system*. Nordin *et al*. also found that UF-SEC isolated and UC isolated EVs show similar patterns in GO term enrichment analysis^9^. However, they did not investigate whether the EV enrichment pattern was specific in terms of differing from the enrichment pattern of free secreted protein.

Another aim of this study was to compare the functional effects of UF-SEC EVs and UC EVs and to investigate whether our UF-SEC protocol can be used to discriminate between the functional effects of EVs and of free secreted protein. The 10 kDa ultrafiltration cut-off is anticipated to equally concentrate EVs and small secreted proteins. After separation using SEC, this should provide well-matched EV and protein concentrates for comparative functional analyses. We found that only UF-SEC protein fractions, but not UF-SEC EVs or UC EVs from cigarette smoke-exposed bronchial epithelial cells induce IL-8 release from naïve bronchial epithelial cells. In contrast, UF-SEC EVs and UC -EVs, but not UF-SEC protein promoted adhesion of THP-1 monocytes to endothelial cells. Thus, UF-SEC is an ideal method for directly comparing the biological effects of EVs to those of the non-EV associated secretome. Concerning the comparison of the two EV isolation techniques, results were similar for UF-SEC EVs and UC EVs. This is in contrast to findings of Mol *et al*. who reported a higher functionality of SEC-isolated EVs^10^. However, while the amplitude of the THP-1 adhesion to endothelial cells was similar for UF-SEC EVs and UC EVs, variability was higher for the UC EVs, leading to a higher p-value and less discriminative power in the statistical analysis. While it is premature to generalize this observation, the higher variability may be caused by the presence of aggregated EVs in the UC EVs.

When studying EVs in conditioned media of cell monocultures, it is obvious from what cell type the EVs are derived. However, in translational studies, EVs are investigated in complex body fluids such as plasma. A major challenge is to determine the origin of these EVs. Therefore, one of the side aims of this study was to explore whether BEAS-2B bronchial epithelial cell-derived EVs contain cell type-specific proteins. A similar attempt has previously been made by Hurwitz *et al*. who analysed the proteomic composition of 60 cancer cell lines, including 9 that were derived from the pulmonary epithelium^16^. They proposed a list of 64 potentially lung cancer-specific proteins that were identified in at least one of the 9 lung cancer cell lines, but in none of the other 51 cancer cell lines. None of these 64 proteins was found to be associated with BEAS-2B EVs in our study. However, the 160 proteins that were specifically EV-associated (i.e. identified in UF-SEC EVs and UC EVs but not in UF-SEC protein) were highly enriched for lung-related sites of expression, including bronchoalveolar lavage fluid and several pulmonary epithelial-derived cancer cell lines. Thus, our data suggests that pulmonary epithelial-derived EVs carry a unique protein signature. Such a protein signature may be useful for identifying EVs of pulmonary epithelial origin in complex biological fluids.

The UF-SEC EV isolation protocol described in this study provides several advantages. Here, we demonstrate that the technique can be used for proteomic and functional EV characterization. Moreover, others have shown that SEC also allows discriminating between EV-associated and non EV-associated microRNAs^24^. While we estimate that the consumable costs of our technique are comparable to ultracentrifugation, EV isolation can be performed using a normal table top centrifuge. Thus, research groups that start working with EVs are not confronted with the high costs of purchasing an ultracentrifuge. Moreover, in our hands the EV yield and purity appear slightly superior using UF-SEC compared to UC and the technique provides very robust results when performed by inexperienced experimenters. In contrast, we have experienced that the yield of UC isolations strongly depends on the operator. Increasing the size of the 10 kDa centrifuge filters and/or the SEC columns combined with automated fraction collection would allow scaling up the isolation protocol to obtain large amounts of EVs for clinical studies. However, our technique also bears limitations. The sepharose CL-4B matrix used in our study may not be suitable for isolating EVs from plasma as its low size exclusion limit may impair the resolution between EVs and high density lipoprotein (HDL). Moreover, our method may be unable to resolve EVs from very high molecular weight complexes with similar sizes to HDL, such as extracellular proteasome complexes. Variations of the protocol may be applied for isolating larger EVs from cell culture media or for isolating EVs from body fluids. These may include skipping the 0.22 µm filtration, using a larger molecular weight cut-off for ultrafiltration and using a SEC matrix with a larger size exclusion limit.

In conclusion, this study demonstrates that ultrafiltration combined with SEC is an easy and robust method for isolating EVs with exosome properties from cell culture media. The yield and purity of EVs obtained with this method tended to be superior to ultracentrifugation and were sufficient for proteomic and functional analyses. Moreover, UF-SEC provides a well-matched concentrate of the non-EV associated secretome for differential functional analysis.

## Methods

### Cell culture and cell stimulation

BEAS-2B bronchial epithelial cells (CRL-9609; ATCC, Manassas, VA, USA) were cultured, foetal calf serum (FCS; Lonza, Basel, Switzerland) was depleted of EVs and cigarette smoke extract (CSE) was prepared as described previously^13^. Human THP-1 cells (ACC-16; DSMZ, Braunschweig, Germany) were cultured at 37 °C in RPMI-1640 supplemented with 10% FCS and 1% penicillin and streptomycin. Human umbilical vein endothelial cells (HUVEC, C-12203; PromoCell, Heidelberg, Germany) were cultured at passages 2–8 at 37 °C in endothelial cell growth medium (Lonza) constituted with endothelial supplement mix (Lonza) and 1% penicillin and streptomycin.

For EV-isolations, 4 × 10^6^ BEAS-2B cells were seeded per T75 in RPMI-1640 (Gibco) containing 10% FCS and allowed to attach overnight. After 2 h incubation in reduction medium (DMEM-F12 containing 0.1% EV-depleted FCS), cells were washed twice with phosphate buffered saline (PBS) before 20 ml reduction medium with either 1% (v/v) PBS (vehicle control) or, for functional studies, 1% (v/v) CSE was added. Three T75 flasks and a total medium volume of 60 ml were used per condition for EV isolation by UF-SEC and two flasks (40 ml) for ultracentrifugation (UC) or ultracentrifugation including a PBS wash step (UC-wash). For nano LC-MS/MS, 6 flasks (120 ml) were used for both isolation methods and media were processed in 2 aliquots of 60 ml (UF-SEC) or 3 aliquots of 40 ml (UC).

### EV isolation

After overnight incubation of BEAS-2B cells with 1% (v/v) PBS or CSE, conditioned media were harvested and centrifuged at 4 °C to remove cells and cell debris (15 minutes at 5,000 × g). Next, media were passed through a 0.22 µm filter.

For UF-SEC, media (60 ml) were loaded onto Amicon Ultra-15 Centrifugal Filter Units with Ultracel-10 membrane (MWCO = 10 kDa; Merck Millipore, Billerica, MA, USA) and concentrated to ≤300 µl by repeated centrifugation at 4000 × g. The concentrate was collected, after which the filter membrane was washed with 200 µl PBS, which was added to the concentrate. Where necessary, sample volume was adjusted to 500 µl. Media concentrates were fractionated by SEC as described by Böing *et al*.^7^ with some adaptations. In brief, 500 µl of concentrated media was applied to a 10 ml sepharose CL-4B column (GE healthcare, Little Chalfont, UK) and 24 fractions of 0.5 ml were collected using PBS as an eluent. Protein content of the fractions was determined using the Bradford assay (Bio-Rad, Hercules, CA, USA) according to the manufacturer’s instructions. The EV rich, protein-low fractions (6 until 10 or 11) and the protein- rich, EV-low fractions (14 until 19) were then pooled and concentrated to 250 µl on an Amicon Ultra-4 Centrifugal Filter Unit with Ultracel-10 membrane (MWCO = 10 kDa; Merck Millipore) by centrifugation at 4,000 × g and stored at −80 °C.

Alternatively, 0.22 µl filtered media (40 ml) were centrifuged in an Optima L-90K preparative ultracentrifuge (Beckman-Coulter, Brea, CA, USA) using a fixed-angle Type 70Ti-rotor (Beckman-Coulter) and QuickSeal tubes (Ultra-Clear, 39 ml, Beckman-Coulter) at 40,000 rpm (Average RCF = 117,734 × *g*, k-Factor 133.7) for 2.5 h (optimal speed and duration as determined by Cvjetkovic *et al*.^12^). After ultracentrifugation, supernatant was poured off and the invisible EV-pellet was resuspended either in 200 µl PBS and stored at −80 °C, or in 5 ml PBS for further purification by a second 2.5 h ultracentrifugation step using an NVT90 rotor and QuickSeal tubes (Ultra-Clear, 5.1 ml, Beckman-Coulter) at 40,000 rpm (Average RCF = 110,656 × *g*, k-Factor 48.3). The PBS-washed EV pellet was resuspended in 200 µl PBS and stored at −80 °C.

### Assessment of EV recovery using CD63^+^CD81^+^ bead-coupled flow cytometry

Throughout the EV isolation procedures, samples were taken to determine the efficiency of isolation by CD63^+^CD81^+^ bead-coupled flow cytometry^25^. Unfiltered and 0.22 µm-filtered conditioned media, as well as SEC-fractions and UF flow-throughs were assessed undiluted, while UF concentrates and final EV isolates from the UF-SEC, UC or UC-wash methods were diluted to the volume from which they were isolated. Beads (3.5 × 10^8^ ml^−1^, 4 µM aldehyde/sulphate latex beads 5% (w/v); Thermo Fisher Scientific) were coated with 0.125 mg/ml mouse anti-human CD63 antibody (Clone H5C6; BD Biosciences, San Jose, CA, USA) by overnight incubation in MES buffer and stored at 4 °C in PBS containing 0.1% (m/v) glycine and 0.1% (m/v) sodium azide. Two hundred microliter of each EV sample were incubated overnight with 1 × 10^6^ beads. Beads were then washed twice by centrifugation in PBS- 2% (m/v) BSA (5000×, 5 min). This was followed by staining for 1 h in 50 µl PBS-2% (m/v) BSA containing 0.01 mg/ml PE-labelled mouse anti-human CD81 antibody (Clone JS-81; BD Biosciences). The stained EV-coupled beads were washed twice by centrifugation (5000 × g, 5 min), resuspended in 150 µl PBS and analysed using a BD FACSCanto (BD Biosciences) with FACS Diva V8.0.1 software (BD Biosciences). The quantity of EVs in relative fluorescent units (RFU) was calculated by multiplying the percentage of PE-positive beads with the median fluorescent intensity (MFI) of the positive bead population. For recovery calculations, all measurements were expressed as percentage of the unfiltered conditioned medium. For the flow cytometry stainings in Fig. 2c, beads were alternatively coated with mouse anti-human CD81 (clone JS-81; BD Biosciences) or mouse anti-human CD9 (clone M-L13; BD Biosciences), followed by detection using PE-labelled mouse anti-human CD81 (clone JS-81; BD Biosciences) or mouse anti-human CD9 (clone M-L13; BD Bisosciences), respectively. All volumes and concentrations were maintained identical as for the CD63/CD81 staining.

### Protein determination by Bradford microplate assay

The Bradford microplate assay (Bio-Rad) was performed according to manufacturer’s protocol with some modifications to allow improved sensitivity. The standard curve was a 2-fold serial dilution ranging from 100 µg/ml to 3.125 µg/ml bovine serum albumin (BSA). 50 µl of each standard or protein sample were loaded on a 96-well plate followed by addition of 250 µl/well Bradford reagent. Absorption was measured at 595 nm.

### Western blotting

For Ponceau S staining and Western blotting, the 24 SEC fractions were precipitated with acetone containing 10% (v/v) trichloric acid (TCA) and 20 mM 1,4-dithiothreitol (DTT). The protein pellets were resuspended in 30 µl XT sample buffer (Bio-Rad) containing 5 M urea and cOmplete™ protease inhibitor cocktail (Roche Life Science, Penzberg, Germany) at the concentration recommended by the manufacturer. Fifteen microliter were loaded onto a 12% running gel for sodium dodecyl sulphate polyacrylamide gel electrophoresis (SDS-PAGE) according to standard protocol. Transfer to an Amersham^TM^ Protran^TM^ 0.2 µm nitrocellulose membrane (GE Healthcare) was performed by wet blotting and detection of general protein signal by Ponceau S staining. Next, non-specific binding sites were blocked using 5% (w/v) BSA in tris-buffered saline, followed by immunostaining using one of the following antibodies: mouse anti-human CD63 (clone H5C6, BD Biosciences, 1000 × diluted), mouse-anti human CD81 (clone JS-81; BD Biosciences, 1000 × diluted), mouse anti-human MFGE8 (R&D Systems, clone 278918, 500× diluted) or mouse anti-human HSC70/HSP70 (Enzo Life Sciences, clone N27F3-4, 500 × diluted). A detailed description of the staining protocol, as well as of the image acquisition and processing is provided in the supplementary methods, along with uncropped images of the Ponceau S and immunostaining (Supplementary Figure S1).

### Cryo transmission electron microscopy (cryo-TEM)

Five microliter of UF-SEC EV isolates was applied to a glow-discharged holey carbon grid. The grid was incubated for 4 minutes at room temperature before blotting against filter paper to leave only a thin film spanning the grid holes. The sample was kept at 95% humidity before plunge-freezing in liquid ethane using a Vitrobot (FEI, Eindhoven, The Netherlands). The vitreous sample films were transferred to a Tecnai T12 Spirit microscope (FEI) using a Gatan cryo-transfer. The images were taken at 120 kV with a 4,096 × 4,096 pixel CCD Eagle camera (FEI) at a temperature between −170 °C and −175 °C and using low-dose imaging conditions.

### Tuneable resistive pulse sensing (TRPS)

TRPS was performed using a qNano Gold with Izon Control Suite 3.2 Software, an NP150 nanopore and SKP200 calibration beads (Izon, Chirstchurch, New Zealand). Cell-depleted unfiltered media and EV isolates were analysed after a single freeze-thaw cycle at −80 °C. Media were diluted 2-fold and EV isolates up to 20-fold in Solution Q (Izon). To improve comparability between measurements, the following settings were used: For UF-SEC media and isolates, the stretch of the NP150 nanopore was adjusted to obtain a relative particle size of approximately 0.65 for the SKP200 calibration beads. A concentration fraction from 80 to 250 nm was applied to these measurements. For UC and UC-wash media and isolates, the relative particle size for the SKP200 calibration beads was adjusted to 0.25, as smaller stretches resulted in pore obstruction. A concentration fraction from 110 to 300 nm was applied to these measurements. For all measurements, the voltage was adjusted to obtain a current between 120 and 130 nA. EV measurements of the dilute media were stopped after 10 minutes, while measurements of the more concentrated EV isolates were stopped after detecting 500 blockades.

### Proteomic analysis and database search

UF-SEC EVs (n = 5), UC-EVs (n = 3) or UF-SEC protein (n = 3; 500 µl per sample for all sample types, isolated from 120 ml of cell culture media) were precipitated by overnight incubation at −20 °C with 1.5 ml acetone containing 10% (v/v) TCA and 20 mM DTT followed by centrifugation for 10 min at 16,000 × g and 4 °C. After washing with ice cold acetone, the protein pellets were resuspended in 30 µl 50 mM ammonium bicarbonate (ABC; Sigma-Aldrich) containing 5 M urea, and the protein concentration was determined using Bradford assay (Bio-Rad). To 5 µg of protein per sample, DTT (20 mM) was added and incubated at room temperature for 45 minutes. Iodoacetamide (40 mM) was then added to alkylate for 45 minutes at room temperature until quenching by a second addition of DTT. A mixture of trypsin/lysozyme C (2 µg) was added and digestion was performed at 37 °C for 2 hours. The mixture was diluted with ABC without urea and incubation was continued at 37 °C for 18 hours. The digestion mix was briefly centrifuged and the digest/peptide mixture diluted fourfold for nano-LC MS/MS analysis. A nanoflow HPLC instrument (Dionex ultimate 3000) was coupled in-line with a Q Exactive mass spectrometer (Thermo Fisher Scientific) with a nano-electrospray Flex ion source (Proxeon, Thermo Fisher Scientific). Five microliter of the digest/peptide mixture was loaded onto a C18-reversed phase column (Acclaim PepMap C18 column, 75-μm inner diameter × 15 cm, 2-μm particle size; Thermo Fisher Scientific). The peptides were separated with a 90 min linear gradient of 4–45% buffer B (80% acetonitrile and 0.08% formic acid) at a flow rate of 300 nl/min.

MS data was acquired using a data-dependent top10 method, dynamically choosing the most abundant precursor ions from the survey scan (250–1250 m/z) in positive mode. Survey scans were acquired at a resolution of 70,000. Dynamic exclusion duration was 30 s. Isolation of precursors was performed with a 4.0 m/z window. Resolution for HCD spectra was set to 17,500 and the Normalized collision energy was 30 eV. The under fill ratio was defined as 1.0%. The instrument was run with peptide recognition mode enabled, but exclusion of singly charged and charge states of more than five.

The data was analysed using Sequest HT Proteome Discoverer 2.1 search engine (Thermo Fisher Scientific), against the Uniprot database. The false discovery rate (FDR) was set to 0.01 for proteins and peptides, which had to have a minimum length of 6 amino acids. The precursor mass tolerance was set at 10 ppm and the fragment tolerance at 0.2 Da. One miss-cleavage was tolerated, oxidation of methionine was set as a dynamic modification and carbamidomethylation of cysteine residues was a fixed modification. Proteins with a HT score >10 were considered as being identified with high confidence.

### Functional assays

For assessing IL-8 release, 5 × 10^4^ BEAS-2B cells/well were seeded on 48-well plates and allowed to attach overnight. Cells were washed twice with PBS before stimulation with 2 × 10^8^ EVs/ml obtained by UF-SEC, the volume-matched UF-SEC protein, or 2 × 10^8^ UC EVs. For cell-free controls, the same dilution of UF-SEC protein was incubated in cell-free wells of the 48-well plate. After 48 h at 37 °C, media were harvested and depleted of cells and cell debris by centrifugation (5,000 × g, 5 min). IL-8 concentrations were assessed using ELISA (Ready-SET-Go; eBioscience, San Diego, USA) according to the manufacturer’s instructions.

For flow chamber adhesion, HUVEC were seeded in 35-mm, 30 µg/ml collagen-coated dishes and incubated with 7.5 × 10^7^ UF-SEC EVs/ml, volume-matched protein isolates, or 7.5 × 10^7^ UC EVs/ml for 72 h. THP-1 cells were labelled with 1 μM green fluorescent nucleic acid stain (Syto 13) for 30 min at 37 °C, washed and perfused in Hank’s buffer at pH 7.45, containing 10 mM HEPES, 3 mM CaCl_2_, and 2 mM MgCl_2_ and 0.2% human albumin for 2–6 min minutes at 3 dynes/cm^2^. Adherent fluorescent cells were manually counted in >6 fields and expressed as cells/mm^2^.

### Data analysis

All data are composed of at least three independent experiments. Data were analysed with Graphpad Prism 5.03 for Windows (GraphPad Software, Inc., La Jolla, CA, USA). Due to the small sample size, the data was analysed using the non-parametric Mann-Whitney test where two groups were compared and the non-parametric Kruskal-Wallis test followed by Dunn’s posthoc test where more than two groups were compared. Unless indicated otherwise, graphs depict each individual data point and lines indicate the medians. P-values ≤ 0.05 were considered statistically significant and are indicated in the graphs as reported by the analysis software: *p 0.01–0.05, **p 0.001–0.01 and ***p < 0.001. Functional enrichment analysis was performed using the hypergeometric test in the FunRich software version 2.1.2^26^.

### Data availability

All data generated and analysed during this study are included in this article and its Supplementary Information files. The raw data of the nano LC-MS/MS analyses have been deposited to the public database ProteomeXchange (Project number: PXD006738).

## Electronic supplementary material

Supplementary Material

Supplementary Table S1
