# Supplementary material for: Ultrafiltration combined with size exclusion chromatography efficiently isolates extracellular vesicles from cell culture media for compositional and functional studies
Source: Sci Rep. 2017 Nov 10;7:15297. doi: 10.1038/s41598-017-15717-7 (PMC5681555; doi:10.1038/s41598-017-15717-7)
Supplement: Supplementary file 1 — Supplementary Material [file 41598_2017_15717_MOESM1_ESM.pdf]

SUPPLEMENTARY MATERIAL

Ultrafiltration combined with size exclusion chromatography efficiently isolates extracellular vesicles from cell culture media for compositional and functional studies

Birke J. Benedikter[1,2], Freek G. Bouwman[3], Tanja Vajen[4], Alexandra C. A. Heinzmann[4], Gert Grauls[1], Edwin C. Mariman[3], Emiel F. M. Wouters[2], Paul H. M. Savelkoul[1,5], Carmen Lopez-Iglesias[6], Rory R. Koenen[4], Gernot G. U. Rohde[2], Frank R. M. Stassen[1,+]

1. *Department of Medical Microbiology, NUTRIM School of Nutrition and Translational Research in Metabolism, Maastricht University Medical Center, PO box 5800, 6202AZ Maastricht, The Netherlands*
2. *Department of Respiratory Medicine, NUTRIM School of Nutrition and Translational Research in Metabolism, Maastricht University Medical Center, PO box 5800, 6202AZ Maastricht, The Netherlands*
3. *Department of Human Biology, NUTRIM School of Nutrition and Translational Research in Metabolism, Maastricht University Medical Center, PO box 5800, 6202AZ Maastricht, The Netherlands*
4. *Department of Biochemistry, Cardiovascular Research Institute Maastricht (CARIM), Maastricht University, Maastricht, The Netherlands, PO box 616, 6200 MD Maastricht, The Netherlands*
5. *Department of Medical Microbiology & Infection Control, VU University Medical Center, Van Boechorststraat 7, 1081BT Amsterdam, The Netherlands*
6. *Microscopy Core Lab, M4I Nanoscopy division, FHML, Maastricht University, Universiteitssingel 50, G0.201, 6229 ER Maastricht, the Netherlands*

+ Corresponding author:

Frank Stassen

Maastricht University Medical Center

Department of Medical Microbiology

PO box 5800

6202 AZ Maastricht

The Netherlands

Tel: +31 43 38 74663

Fax: +31 (0)43 3876643

f.stassen@maastrichtuniversity.nl

Supplementary Methods

Immunostaining and imaging equipment and settings

After membrane transfer, the presence of protein, including the unstained low range SDS-PAGE standard (Bio-Rad) was revealed by Ponceau S staining and an image was recorded. For immunoblots where unstained SDS-PAGE standards were used, the molecular weight standard bands were marked with a pen before destaining. The membrane was blocked overnight at 4°C with 5 % BSA (m/v) in tris-buffered saline (TBS). Next, the membrane was incubated for 2 h at room temperature with one of the following antibodies: mouse anti-human CD63 (clone H5C6, BD Biosciences, 1000x diluted), mouse-anti human CD81 (clone JS-81; BD Biosciences, 1000x diluted), mouse anti-human MFGE8 (R&D Systems, clone 278918, 500x diluted) or mouse anti-human HSC70/HSP70 (Enzo Life Sciences, clone N27F3-4, 500 x diluted). For all primary antibodies, this was followed by 1 h incubation with a polyclonal HRP-labelled rabbit anti-mouse antibody (1:1000; DAKO). Staining was revealed using chemiluminescence-peroxidase substrate-3 (Sigma-Aldrich). Images were recorded using a ChemidocTM XRS+ System (Bio-Rad) with the software Quantity One V 4.6.7 (Bio-Rad). For chemiluminescence recordings, the Chemi Hi Sensitivity mode was used (2x2 binning, no light, 2x gain), the iris was opened completely and the exposure time was set between 15 and 120 s according to the signal strength. The Ponceau S staining (uncropped in Supplementary Figure 1A, cropped in Figure 2C) and marker bands were recorded with custom settings (2x2 binning, Epi white illumination, 2x gain) using the auto exposure time function. The multichannel viewer of the software was used to make an overlay of the chemiluminescence (green) and light signal (red). Using the software Irfanview V 4.25, the overlay image was then converted to gray scale, followed by a conversion to the negative in order to obtain dark bands on a light background. Uncropped immunoblots are shown in Supplementary Figure 2. These images were cropped in order to show only the relevant bands in Figure 2E.

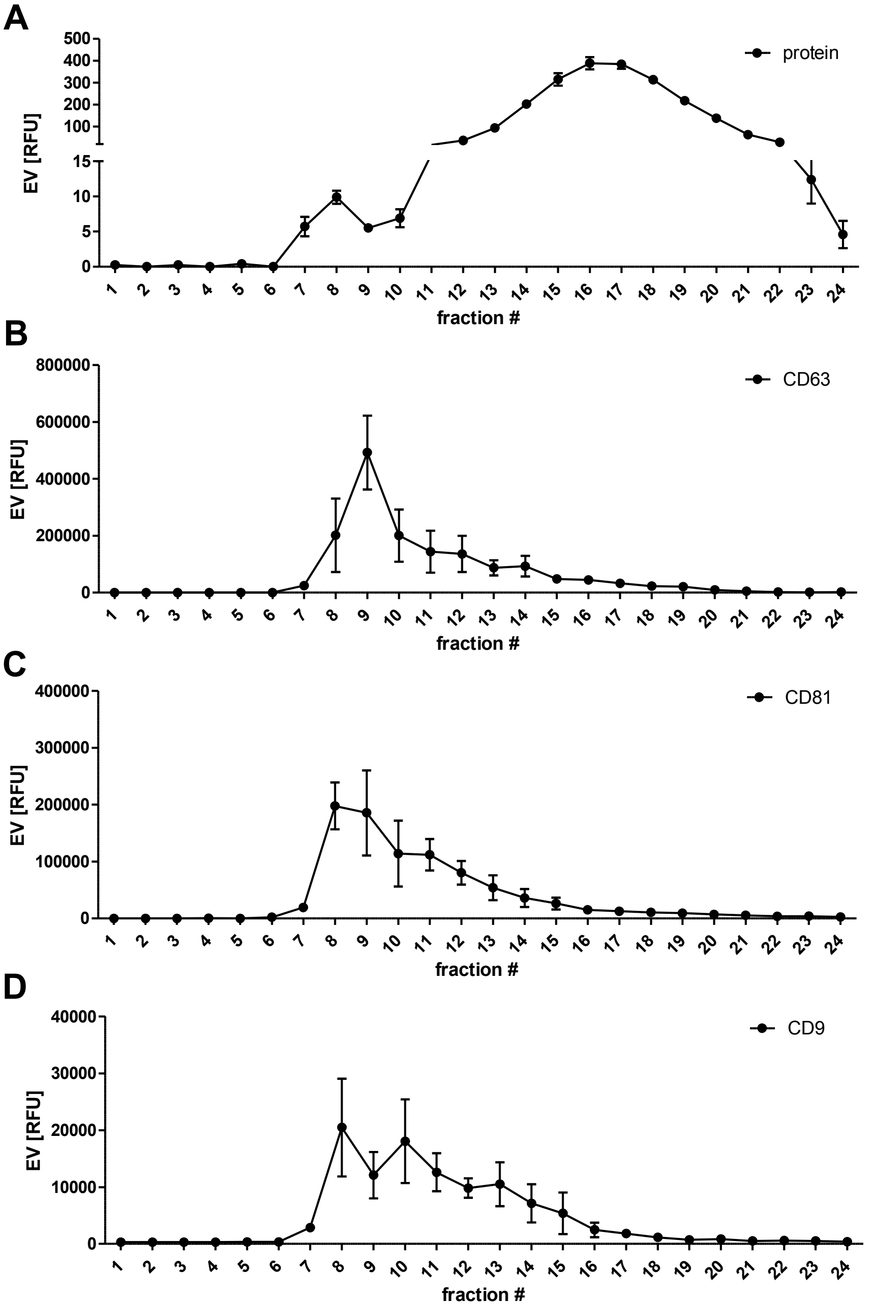

Supplementary Figure S1 – Protein and EV elution in the 24 SEC fractions according to Bradford assay and bead-coupled flow cytometry. The graphs shows the means ± standard error of the mean for 4 independent experiments. (A) The protein concentration according to Bradford microplate assay that was adapted for improved sensitivity. (B) EVs detected by bead-coupled flow cytometry using anti-CD63-coated beads and PE-labelled anti-CD63 detection antibody. (C) EVs detected by bead-coupled flow cytometry using anti-CD81-coated beads and PE-labelled anti-CD81 detection antibody. (D) EVs detected by bead-coupled flow cytometry using anti-CD9-coated beads and PE-labelled anti-CD9 detection antibody

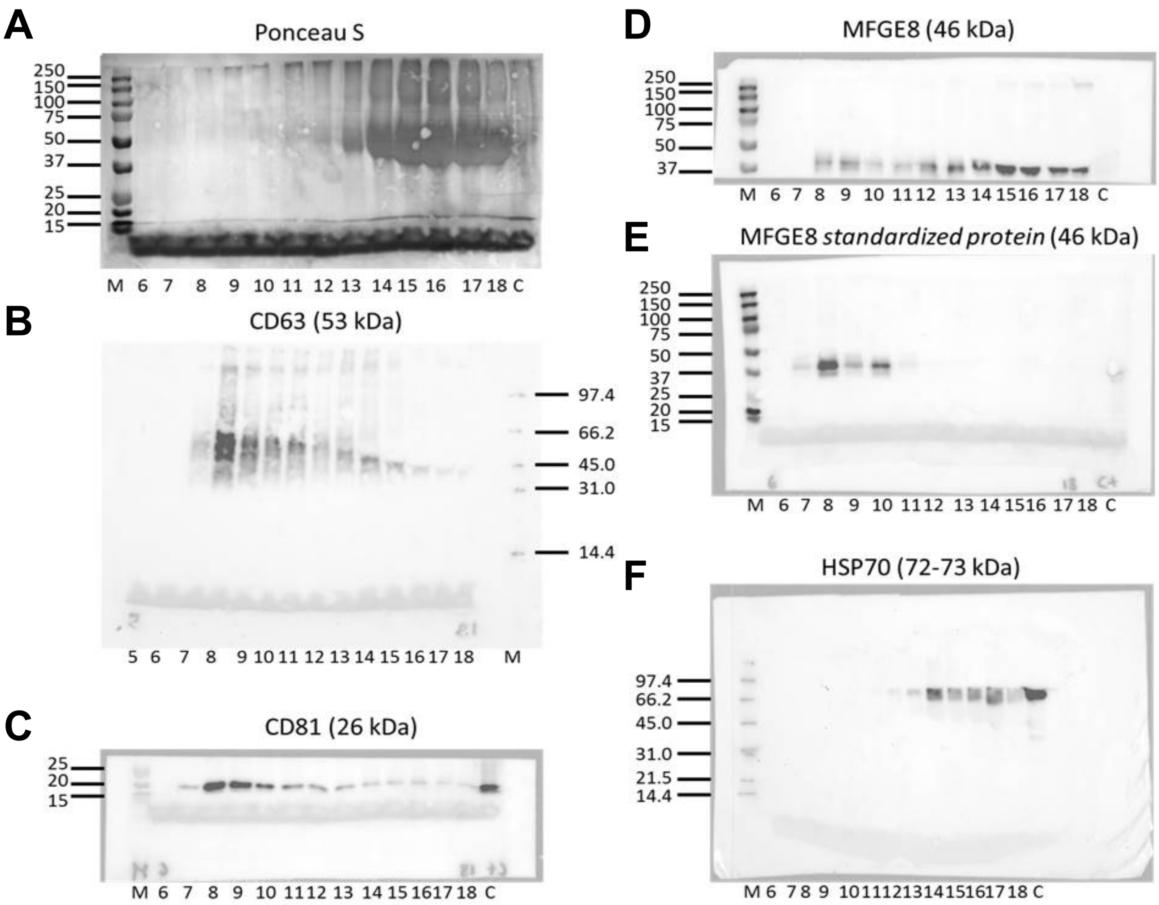

Supplementary Figure S2 – Uncropped images of the stainings in Figure 2E of the main article. (A) Ponceau S staining, (B) CD63 immunoblot, (C) CD81 immunoblot, (D) MFGE8 immunoblot, (E) MFGE8 immunoblot with a standardized amount of protein per fraction (3 µg) and (F) HSP70 immunoblot. The numbers below the images indicate which SEC fraction was loaded into the respective lane. The lane where the molecular weight marker was loaded is marked with an M and the lane in which a BEAS-2B cell lysate was loaded as positive control is marked with a C. Between brackets, the predicted molecular weight of each protein according to the antibody manufacturer is given. For Figures A, C, D and E Precision Plus Dual Color Standard #161-0374 (Bio-Rad) was used, whereas Unstained SDS-Page standards, low range #161-0304 was used for Figures B and F

Supplementary Table S2 – overview over Rab GTPases that were identified by nano LC-MS/MS in UF-SEC EVs, UC EVs and UF-SEC proteins. Proteins identified with a Score Sequest HT >10 were considered as identified with high confidence. In this table, proteins identified with a Score <10 are also included for completeness. The number of peptide spectral matches (#PSM) gives an indication of the relative protein abundance

**EV marker proteins**	**Gene symbol**	**UF-SEC EVs**		**UC EVs**		**UF-SEC protein**	
		identified	#PSM	identified	#PSM	identified	#PSM
Ras-related protein Rab-1A	RAB1A	Score <10	3	No	0	No	0
Ras-related protein Rab-5C	RAB5C	Score<10	2	No	0	No	0
Ras-related protein Rab-7a	RAB7A	Yes	5	Score <10	2	No	0
Ras-related protein Rab-8B	RAB8B	Score <10	3	Yes	5	No	0
Ras-related protein Rab-10	RAB10	Score <10	4	Yes	6	No	0
Ras-related protein Rab-35	RAB35	yes	6	yes	5	no	0
